# The nursing care of people with class III obesity in an acute care setting: a scoping review

**DOI:** 10.1186/s12912-021-00760-7

**Published:** 2022-01-28

**Authors:** Beverley Ewens, Vivien Kemp, Amanda Towell-Barnard, Lisa Whitehead

**Affiliations:** School of Nursing and Midwifery, Edith Cowan University, 270 Joondalup Drive, Joondalup, WA 6027 Australia

**Keywords:** Class III obesity, Nursing care, Acute care settings, Bariatric, Scoping review

## Abstract

**Background:**

Patients with Class III obesity pose unique challenges to health care staff and organisations. Care requirements of this population are unique and require specialised equipment and knowledge to meet these needs, maintain the quality of care, as well as the safety of patients and staff.

**Aim:**

To synthesise the evidence on the nursing care of Class III obese patients in acute care settings.

**Methods:**

A scoping review informed by JBI. CINAHL Plus, Medline, Scopus, Proquest Central, Web of Science and Embase were searched for primary research articles about the nursing management of people classified as Class III obese in acute care. Methodological quality of included studies was assessed; data extracted and synthesised into themes.

**Results:**

Fourteen studies were included in the review. The synthesis generated three themes: Access to equipment, knowledge and training, patient care, and opportunities to improve care.

**Conclusions:**

A paucity of high-quality evidence informs the nursing care of people with Class III obesity in acute care. Access to appropriate equipment dominated the findings of this review. Adequate provision of equipment and education on its use are required. Education to promote engagement with patients, adapting clinical practice and promotion of self-care could improve care and outcomes.

**Supplementary Information:**

The online version contains supplementary material available at 10.1186/s12912-021-00760-7.

## Introduction

Obesity is a complex psychosocial construct which is strongly linked to health and wellbeing, and is an important predictor of mortality and morbidity [1] including diabetes, heart disease and certain cancers [2] . The incidence of obesity is increasing worldwide, with over 650 million adults classified as obese in 2016 and 1.9 billion as overweight [3]. Obesity is not confined to developed countries, but is also an emerging health concern in many developing countries including Bangladesh [4], India [5] and Saudi Arabia [6]. There is also an impact on health organisations, as hospital admissions related to obesity as either a primary or secondary diagnosis are increasing [7]. The classification of obesity however, varies within the literature and is further complicated by the use of the term ‘bariatric’ [8]. Discrepancies in definitions and perceptions of obesity have been acknowledged, particularly in children [3, 9]. Body Mass Index (BMI) remains the most frequently used measure of classification. A BMI of > 30 kg/m^2^ signifies obesity and the World Health Organization (WHO) [3] have further categorised obesity into three sub classes with Class III categorised as the highest level of obesity.

The health and socioeconomic impacts of obesity on individuals and health care systems can be significant. In young and middle aged adults, obesity is associated with lower educational attainment [10], development of comorbidities [11, 12] including cardiovascular disease, musculoskeletal disorders and some cancers [3], increase in disability [13] and overall reduction in life expectancy [14, 15]. Obesity of any classification can increase the complexity of clinical care including mobilisation, skin care and perioperative management [16–18].

The increasing number of hospital admissions of people living with Class III obesity and the associated complexity of caring for them, increases demands on health care facilities as well as presenting unique challenges in relation to nursing care requirements. Patients with obesity often present with comorbid conditions which also complicates their care requirements [19]. There are particular risks to nurses when caring for patients with obesity particularly when they are acutely ill and require assistance; it is well recognised that the risks of musculo-skeletal injury in nursing staff is proportional to the weight of patients and the techniques used [20, 21]. However, there remains a lack of evidence which identifies effective interventions to address the issue of muskulo-skeletal injuries in nurses within this context [22]. Patients with obesity are also at risk of discomfort and injury, both physical and psychological in relation to moving and handling if this is not conducted expertly and with the appropriate equipment, knowledge and skills [23]. It is evident that patients with obesity pose unique care challenges to those nurses who care for them, including pain management [24] and wound management which can necessitate the need for more complex wound management strategies to promote healing [18] and the maintenance of skin integrity [25]. Wound management in patients with obesity is further complicated by a lack of an evidence base, which has been identified as a particular issue [26] but also in many other aspects of care including patient centred communication [27, 28], mobilisation [29], minimisation of pressure ulcers [30], cardiopulmonary resuscitation [31] and respiratory care [16].

This review will therefore synthesise the evidence on the nursing care of people classified as Class III obese in the acute, non-critical care settings, to explore best practice, issues and challenges from the literature.

## Objectives

### Review question

What evidence guides the nursing care of people classified as Class III obese in acute care settings?

### Review objectives

To synthesise the evidence on the nursing care of Class III obese patients in acute care settings.

## Methods

A scoping review was conducted informed by the JBI process [32] and the PRISMA Extension for Scoping Reviews (PRISMA-ScR) [33] was utilised as a framework for this review. Scoping reviews are becoming increasingly more widespread to inform decision making through an examination of the literature on a certain topic [34]. They are considered to be a valid approach and can be used for a variety of reasons [35] including when a systematic review is unable to meet the chosen objectives [36]. Scoping reviews include evidence of any methodology and extend to the inclusion of other evidence such as policies and guidelines [34]. A scoping review can not only answer broader questions than can be answered in a systematic review but also provide an indication of the scope of available evidence, including that which is emerging [36]. In this instance, the researchers were not aiming to provide evidence to inform clinical practice by a synthesis of the evidence to answer a specific question, but to identify and analyse gaps in the knowledge base in relation to the nursing care of patients with Class III obesity within acute care settings [36].

### Eligibility criteria

This review considered primary research studies, published in English, involving participants aged 18 years and over and classified as Class III obese. Studies were included if they reported on the nursing care of people classified as Class III obese within acute care settings, using either qualitative, quantitative or mixed method approaches. Grey literature was not included as we were focusing on the published evidence which informs the development of policies and guidelines. Policies and guidelines which inform practice are not readily available without contacting health service providers, which was not feasible or representative of the range of policies and guidelines available. We were also keen to bring attention to the volume and quality of primary research studies in this important area.

### Information sources

A logic grid was constructed to guide the search strategy (Supplementary Material Table A). A three-step search strategy was employed commencing with an initial search of MEDLINE and CINAHL Plus to identify key words and index terms, followed by a second search across all databases using the identified terms. Thirdly, the reference lists of all 146 identified reports and articles were searched for additional studies. The timeframe from 1980 to 26/07/2018 was chosen, because of the proliferation of interest and associated publications within the context of patients with obesity during this timeframe. Literature searches were conducted between June–August 2017 and again in October 2021.

### Search

The search included the following electronic databases:

CINAHL +, Medline, Scopus, Proquest Central, Web of Science and Embase. The keywords used were: Nursing care, patient care, best practice care, hospital care combined with the terms morbidly obese, and morbid obesity. Boolean exact phrase searching was used in conjunction with mesh terms for obesity, morbid including truncation terms morbid* and obes*, with AND/OR (See Supplementary Material Table A for first search in CINAHL).

### Selection of sources for evidence

This scoping review considered both experimental and quasi-experimental study designs including randomized controlled trials, non-randomized controlled trials, before and after studies and interrupted time-series studies. In addition, analytical observational studies including prospective and retrospective cohort studies, case-control studies and analytical cross-sectional studies were considered for inclusion. This review considered descriptive observational study designs including case series, individual case reports and descriptive cross-sectional studies for inclusion.

Qualitative studies were considered that focused on qualitative data including, but not limited to, designs such as phenomenology, grounded theory, ethnography, qualitative description, action research and feminist research. Text, systematic reviews, reviews and opinion papers were not be considered for inclusion in this scoping review.

**Fig. 1:**
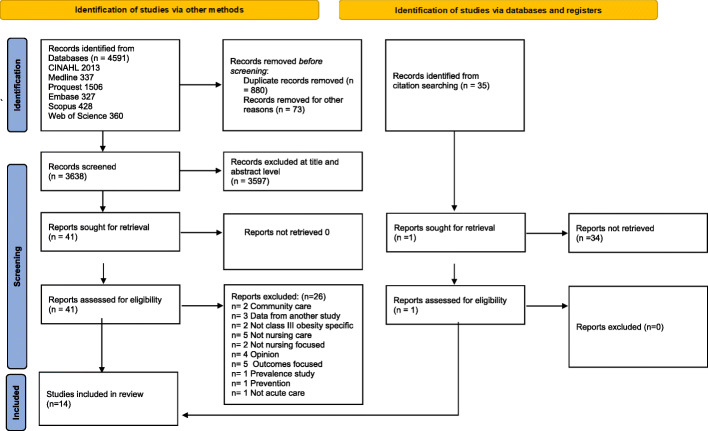
PRISMA Flow Diagram [37]

Studies were excluded if they explored the nursing care of Class III obese patients in critical care areas, perioperative care, perinatal care, and in the community. Studies from these areas were excluded as these are highly specialised areas, where patients have unique needs in relation to their presentation and level of acuity. Potentially, the staff in these areas are more familiar with caring for patients with obesity and are better placed to care for them. This would in particular be relevant in the perioperative and critical care settings where higher staff to patient ratio is routine, equipment is more readily available, and staff are educated in care requirements. Further exclusions included a focus on patient outcomes without reference to nursing care, and studies reporting prevalence of obesity (Fig. 1).

### Data charting process

Data from the eligible studies were charted using a standard charting tool developed and tested by the study team specifically for this study (Table 1). The tool captured the relevant information about the studies including data pertinent to nursing care, and which were extracted from each article by all authors. A data extraction tool was developed by the researchers, data added to the tool and themes generated from it. Any disagreements were resolved by discussion to reach consensus on the themes and subthemes. Due to the heterogeneity of the studies, a meta–analysis could not be completed therefore a synthesis was conducted. The extracted data included details about the purpose and setting of the study, the study population and main findings. No missing or unclear information was found (Table 1).

**Table 1:** Critical appraisal of included studies

First Author and date	Q1	Q2	Q3	Q4	Q5	Q6	Q7	Q8	Q9	Q10
**JBI Critical Appraisal Checklist for Case Reports**										
Broome, CA, 2015 [38]	Y	Y	Y	Y	Y	Y	Y	Y		
Ecklund, MM, 2004 [39]	Y	Y	Y	Y	Y	Y	Y	Y		
Holland, DE, 2001 [40]	Y	Y	Y	Y	Y	Y	Y	Y		
Palmer, R. 2009	Y	Y	U	N	Y	Y	N	Y		
**JBI Critical appraisal Checklist for Descriptive/Case Series**										
Booth CMA, 2011 [41]	N	Y	Y	Y	U	U	U	Y	Y	
Drake, DJ, 2008 [42]	N	Y	Y	Y	U	U	Y	Y	Y	
Gardner, L A, 2013a [43]	N	Y	Y	Y	U	U	U	Y	Y	
Gardner, LA, 2013 [44, 45]	N	Y	Y	Y	U	U	U	Y	Y	
Gardner, LA, 2013b [46]	N	Y	Y	Y	U	U	U	Y	Y	
Hignett, S, 2007 [47]	N	Y	Y	Y	U	U	U	U	Y	
Rose, MA, 2007 [48]	Y	Y	Y	Y	Y	U	U	Y	Y	
**JBI Critical Appraisal Checklist for Interpretive and Critical Research**										
Drake, DJ, 2005 [49]	Y	Y	Y	Y	Y	N	N	Y	U	Y
Rose, MA, 2010 [50, 51]	y	Y	Y	Y	Y	Y	N	Y	Y	Y
**JB**I **Critical Appraisal Checklist for Analytical Cross Sectional Studies**										
Dockrell, S, 2021 [52]	Y	Y	Y	U	Y	N	Y	Y		

Y = Yes, N = No, U = Unclear

### Data items

We abstracted data on the characteristics of the article e.g. methodology, country of origin and health care setting. We also abstracted data in relation to the purpose of the study, the study population and their demographic profile. The specific data items abstracted related to the nursing care of people with Class III obesity, including all aspects of nursing care and classification of obesity across studies.

### Critical appraisal of individual sources of evidence

Quality appraisal of chosen articles is not a requirement of a scoping review [34], however, we chose to undertake this to add further rigour to the study and in particular as the focus of the review was the evidence which underpins nursing care. The care of patients living with Class III obesity is an increasingly common and unique area of practice and as such we felt it important to highlight the quality of evidence currently available. Three independent reviewers assessed the remaining 14 articles that met the inclusion criteria for methodological validity, using the relevant JBI critical appraisal checklist (See Supplementary for JBI Check lists). All authors contributed to the assessment of papers and critical appraisal process, and any disagreements were resolved though group discussion. Seven articles were appraised using the JBI Critical Appraisal Checklist for Descriptive/Case Series studies, four articles using the Checklist for Case Reports, two articles using the Checklist for Interpretive & Critical Research and one article using the Checklist for Analytical Cross Sectional Studies. Articles were included if they scored Yes to 4 or more questions (Table 1). Only one study [53] of the four case studies did not identify any adverse events; the condition of the patient was also unclear. All other case reports met the criteria in full. There were seven descriptive/case series included in the review, five of which [41, 43, 44, 46, 47] lacked rigour around clear reporting of participants and complete inclusion of participants. The three remaining studies omitted to situate the researcher culturally or theoretically [49] or identify the influence of the researcher on the study [49, 50]. The majority of sources of evidence were low level evidence, predominantly case studies and retrospective analysis of data.

### Synthesis of results

Analysis of the findings was undertaken by comparing and contrasting the findings across all studies to identify common concepts and themes that were then iteratively grouped firstly, into subordinate and finally into four superordinate themes [34] (Table 2). (Table 2). We then undertook an aggregative approach to the narrative synthesis of the findings to determine how they related to each other across studies.

**Table 2:** Themes generated from the findings

Themes		Authors														
Superordinate Themes	Subordinate themes	Booth et al., 2011 [41]	Broome et al., 2015 [38]	Dockrell & Hurley, 2021 [52]	Drake et al., 2005 [49]	Drake et al., 2008 [42]	Ecklund & Kurlak, 2004 [39, 54]	Gardner & Gibbs 2013 [44, 45]	Gardner & Pagano, 2013a [43]	Gardner & Pagano,2013b [46]	Hignett et al., 2007 [47]	Holland et al., 2001 [40]	Palmer, 2004 [53]	Rose et al., 2007 [48]	Rose et al., 2010 [50]	Frequency
*Access, knowledge and training related to equipment*	Appropriate equipment available		X		X	X	X	X	X	X	X	X	X		X	11
	Appropriate equipment unavailable	X	X	X	X	X	X	X		X	X	X	X		X	11
	Equipment failure/malfunction	X	X					X			X				X	5
	Staff knowledge about how to use equipment			X				X			X	X				3
	Weight capacity of equipment identified and known		X					X			X	X				4
Patient Care	Circulation issues						X									1
	Elimination (and personal hygiene) needs		X		X		X									3
	Gait and mobility issues	X	X		X		X			X	X	X	X	X	X	10
	Maintaining patient comfort and dignity	X	X				X	X			X	X	X		X	8
	Maintaining patient safety	X	X		X			X	X	X	X	X	X		X	10
	Nutritional assessment to insure adequate caloric intake		X				X									2
	Pain and symptom management		X								X					2
	Patient care plan (daily routine)		X				X		X		X	X				5
	Patient education about their own self-care		X				X									2
	Patient’s psychosocial care and needs		X		X		X					X				4
	Respiration issues		X				X					X	X			4
	Skin integrity issues identified		X				X			X		X				4
*Opportunities to improve care*	Communication between staff members		X				X	X			X					4
	Nurses’ concerns/attitudes/safety	X	X		X	X					X		X			6
	Patient’s acuity and independence levels determined how challenging nursing patients with class III obesity was		X		X											2
	Protocols available on the care of people living with class III obesity	X	X	X	X		X	X	X	X	X	X	X			10
	Hospital policies and procedures for the care of patients with class III obesity either not in place or not followed	X		X	X			X			X		X			5
	Increased resources needed to care for patients with class III obesity	X	X	X	X	X		X			X	X		X		7
	Infrastructure/facility not retrofitted or lack of space to accommodate necessary equipment (including lifts, floors and doors)	X	X		X		X	X		X	X		X	X		9
	Multidisciplinary/interdisciplinary approach to the care of patients with class III obesity	X	X				X					X	X			5

Three major themes emerged from the synthesis (Table 3). These were access, knowledge and training related to equipment; patient care; and opportunities to improve care.

**Table 3:** Results of Individual Sources of Evidence

Author & date	Study Method	Setting	Purpose	Sample	Outcomes Measured
Booth et al., 2011 [41]	Retrospective Registry Records	United Kingdom, Hospital	To ascertain the number of reported patient safety events involving people with obesity	People living with class III obesity in acute care	Patient safety incidents involving people living with obesity
Broome et al., 2015 [38]	Case study	USA, Hospital	To describe the care of a ‘super’ bariatric patient	A person with class III obesity (*n* = 1), aged 56 years, BMI 73 kg/m2	A description of the complex interdisciplinary care challenges for one patient with class III obesity
Dockrell & Hurley, 2021 [52]	Analytical cross-sectional survey	Ireland, Hospital	To explore frequency, logistics, and barriers of bariatric equipment availability in acute care hospitals	Clinical nurse managers working in acute care settings (*n* = 132), 110 (83.2%) had > 3 years’ experience	Barriers to the provision of care for people with class III obesity
Drake et al., 2005 [49]	Qualitative; focus groups, thematic analysis	USA, Hospital	To investigate nurses’ perceptions of the challenges they face in caring for patients with class III obesity in the acute care setting	Nurses whose role included caring for people with class III obesity (*n* = 17). Three males, 14 females, mean age 38.32 years old and mean nursing experience 13 years. Five participants held a baccalaureate degree, 9 held an associate degree, 2 held a diploma of nursing.	Care challenges faced by nurses when caring for patients with class III obesity
Drake et al., 2008 [42]	Descriptive, survey	USA, Hospital	To determine nurses’ perception of the challenges in caring for people with class III obesity	Members of the National Association of Bariatric Nurses. Nine males 100 females.	Pressure ulcer prevalence in patients with a BMI ≥40 kg/m2 and Braden Scale of ≥16, compared to patients with lower BMI
Ecklund & Kurlak, 2004 [39, 54]	Case study	USA, Hospital	To highlight issues involved in caring for a person who has class III obesity	A person with class III obesity, male 39 years old, BMI 91 kg/m2	Strategies to manage multisystem and organisational issues of managing a patient with class III obesity
Gardner & Gibbs, 2013 [44, 45]	Descriptive, retrospective records review and hospital survey	USA, Hospital	To ascertain the number of reported patient safety events involving people with class III obesity	Patients living with class III obesity in acute care (*n* = 1774)	Part 1: Number of patient safety incidents involving people living with Class III obesity Part 2: Pennsylvania hospitals’ readiness to accommodate patients with class III obesity.
Gardner & Pagano 2013a [43]	Descriptive, retrospective records review and hospital survey	USA, Hospital	To ascertain the reported number of serious skin integrity events involving people with class III obesity	Patients living with class III obesity in acute care (*n* = 1774)	Part 1: Event reports of people living with class III obesity reviewed for skin integrity issues. Part 2: The prevalence of patient skin care protocols for patients with class III obesity
Gardner & Pagano, 2013b [46]	Descriptive, retrospective records review and hospital survey	USA, Hospital	To ascertain the reported number of falls event reports involving people with class III obesity	Patients living with class III obesity in acute care (*n* = 1774)	Part 1: Event reports involving falls in people living with class III obesity Part 2: Hospital state-wide survey about hospital preparedness to care for patients with class III obesity and falls
Hignett et al., 2007 [47]	Descriptive. Mixed methods (focus groups and questionnaire)	United Kingdom, special interest groups	To identify and explore manual handling risks and process planning pathways for patients with class III obesity	Members of the National Back Exchange (NBE) (*n* = 224). Special Interests Group on Bariatrics and The National Ambulance Risk and Safety Forum (NARSF) (*n* = 25)	Manual handling risks and pathway planning for patients
Holland et al., 2001 [40]	Case study	USA, hospital	To use a case report to illustrate care and discharge planning for a patient with class III obesity	A person living with class III obesity in acute care. Male aged 49, BMI 72.6 kg/m^2^	Care and discharge planning requirements
Palmer, 2009	Case study	United Kingdom, hospital	To illustrate the strategies employed to aid the moving and handling of one patient with class III obesity	A person living with class III obesity, female, age and BMI not reported	Requirements for safe moving and handling of a patient with class III obesity
Rose et al., 2007 [48]	Cross-sectional, naturalistic observation	USA, hospital	To compare resource requirements when caring for patients with class III obesity and those who do not have obesity	Nursing staff caring for patients with class III obesity and patients who were not obese in an acute care setting	Resource and safety concerns when caring for patients with class III obesity
Rose et al., 2010 [50]	Descriptive, qualitative, semi-structured interview	USA, professional association members	To examine nurses’ perceptions of safety concerns when caring for patients with class III obesity	Nurses who are members of the National Association of Bariatric Nurses (NABN) (*n* = 19)	Number of adverse events, near misses and out-of-control situations in relation to the care of patients with class III obesity

## Results

### Selection of sources of evidence

The initial search identified 4591 articles from six databases, following removal of duplicates, 3638 articles were screened. Thirty-five records were identified from citation searching, and of those following review at abstract level, one was sought for retrieval. All articles retrieved for full text review (*n* = 41) which were identified from the searching process, were screened by all researchers to ensure consistency in this process. Of these 41 articles, 26 were excluded for the following reasons: community care focused (*n* = 2), data reported from another study (*n* = 3), not Class III obesity specific (*n* = 2), not nursing care (*n* = 5), not nursing focused (*n* = 2), opinion piece (*n* = 4), outcomes focused (*n* = 5), prevalence study (*n* = 1), prevention of obesity (*n* = 1) and not acute care (*n* = 1). This resulted in 14 studies retained for full text review. The articles were  shared between all researchers who  screened the articles independently and compared answers with one other researcher. Any inconsistencies were resolved by a third researcher.

### Characteristics of sources of evidence

The studies’ origins, purpose, design, methods, target group and main findings are presented in Table 4. The number of studies which addressed the particular aspects of nursing care grouped into 25 subordinate themes, ranged from one to 11.

**Table 4:** Definitions of Class III Obesity Within Studies

Definition	Author
No definition	Booth et al., 2011 [41]
	Dockrell & Hurley, 2021 [52]
	Drake et al., 2005 [49]
	Drake et al., 2008 [42]
	Rose et al., 2007 [48]
Class III obese patients have a BMI greater than or equal to 40 or 100 pounds more than their idea body weight	Gardner and Gibbs, 2013 [44, 45]
	Gardner and Pagano, 2013a [46], 2013b [46]
Morbid obesity greater than 100 pounds above desirable weight. Severe obesity BMI greater than or equal to 50 km/m^2^	Ecklund & Kurlak, 2004 [39, 54]
Morbid obesity is a body mass index greater than 40 kg per square meter	Holland et al., 2001 [40]
	Palmer, 2004 [53]
Morbidly obese (BMI > 40), super obese (BMI > 50) and super, super obese (BMI > 60)	Broome et al., 2015 [38]
Morbidly obese patient (BMI > 35)	Rose et al., 2010 [50]
Some definitions were by pre-determined weight, some by predetermined size, others when weight exceeded predetermined value and/or exceeded equipment size	Hignett et al., 2007 [47]

### Critical appraisal within sources of evidence

As discussed, critical appraisal of sources of evidence was undertaken in this review. The JBI critical appraisal tools were utilised to appraise the quality of sources of evidence sourced and are provided in supplementary evidence (Fig. 2). The quality appraisal results for each individual study are detailed in Table 1.

**Figure:**
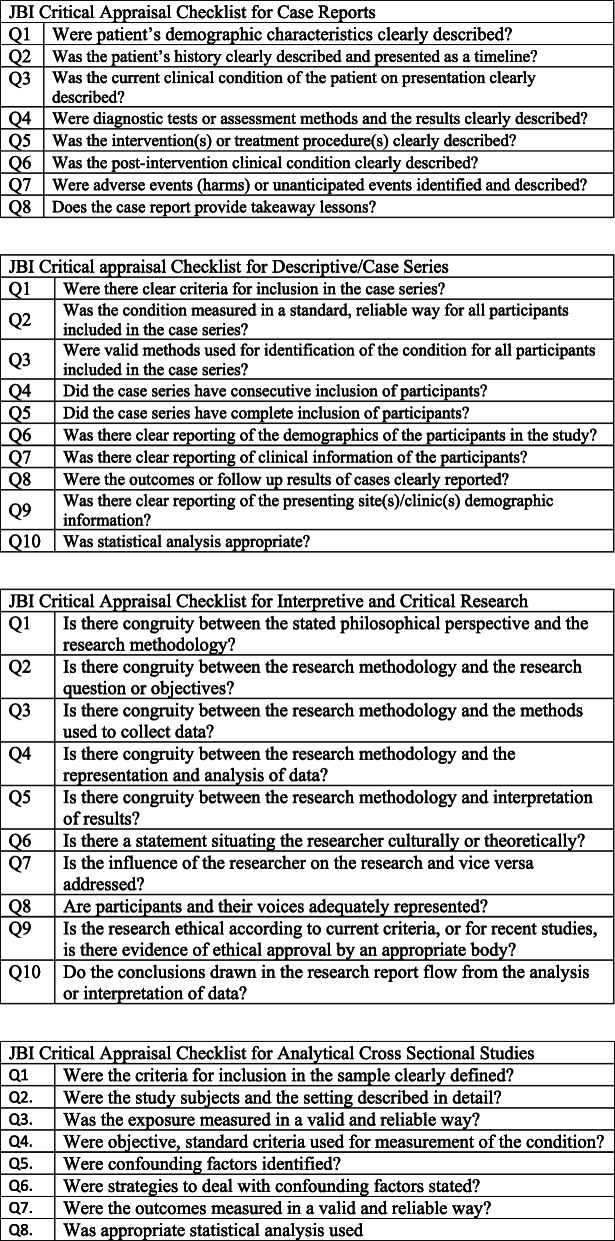
**Fig. 2** JBI Critical Appraisal Tools

### Results of individual sources of evidence synthesis of results

The data from the individual sources is provided in Table 2.

## Discussion

### Summary of evidence

#### Access, knowledge and training related to equipment

Thirty-six findings from 13 studies contributed to this theme. Equipment was identified as a challenge to providing care for people classified as Class III obese and all findings related to either lack of access to appropriate equipment or knowledge and skills to use equipment appropriately. The specific issues raised were accessing appropriate equipment [38, 40, 44, 47, 49], the storage of equipment [49], staff knowledge around how to use equipment [40, 44, 47], patient distress and discomfort through the use of incorrect equipment [38, 41, 53, 55], putting people at risk of harm through the use of inappropriate equipment [38, 41, 47, 49, 51, 53], lack of equipment to measure vital signs [45] and delay in access to rental equipment [44, 47, 49]. Other issues identified were equipment failure or malfunction [41, 51] and, the ability of staff to identify the weight capacity of equipment [40, 45] [40, 45]. Higher levels of staff satisfaction were reported when adequate equipment was available to safely care for the patient [42].

#### Patient care

Forty-two findings from 12 studies contributed to the theme on patient care. Fundamental nursing care was described as becoming more challenging due to the patient’s body habitus, including changing dressings, checking for bowel sounds and heart sounds [49, 51]. Management of respiratory function (including obstructive sleep apnoea, oxygen saturation levels) and skin integrity (local care to wounds, pressure reduction equipment) were also reported [39, 40, 43]. Supporting people to mobilise, and maintaining patient safety were the most frequently reported issues (*n* = 10, 77%), it was noted for example that gait instability [56] and patients overestimating their mobility capacity [38] could pose a threat to patient safety. The need to develop a comprehensive care plan was identified as being vital to assist both staff and patients to anticipate the patient’s individual care needs [40] and to build a therapeutic alliance [38, 54]. Communication was the second most common concern reported by staff in the review. Effective communication between staff and patients was described as essential in promoting a therapeutic relationship [38, 45], and between staff in order to meet patient care needs [42] and communicate needs at handover [38]. The role of nurses in promoting self-care for these patients was also described [38, 54].

#### Opportunities to improve care

Opportunities to improve care was a significant finding from this review with 51 findings from 13 studies contributing to this theme. The majority of studies identified that acute care settings were not designed to care for patients with Class III obesity and accommodate their care needs, particularly in relation to dedicated equipment. Most facilities were retrofitted and not fit for purpose to accommodate patients with Class III obesity. Fifty-one findings from 13 studies contributed to this theme. References were made to specific policies on the care of people classified as Class III obese in the majority of studies. A number of issues were identified in relation to the implementation of policies, procedures and protocols across all aspects of care. This finding related to whether or not there were policies and procedures in place on the one hand, and whether the policies and procedures were followed on the other [47, 52]. This included the benefit of specific discharge planning [38, 40] and evacuation planning [45], as well as the capability to weigh and measure patients on admission to hospital so that the appropriate equipment for each patient was both accessible and available [45, 54]. Other issues identified included staff training and education. This included the benefit of specific discharge planning [38, 40] and evacuation planning [45], as well as the capability to weigh and measure patients on admission to hospital so that the appropriate equipment for each patient was both accessible and available [45, 54]. Other issues identified included staff training and education [38, 41, 44, 47, 53], skin care protocols [38, 40, 44] and the inconsistency in the use of the term ‘bariatric’ [47, 49]. Not all hospitals had established policies relating to the manual handling of people classified as Class III obese [47, 52]. These issues are a concern because Class III obese patients were found to require a greater proportion of staff numbers to care for them, require larger rooms, specialised equipment and other resources in acute care settings compared to other patients [48]  including insufficient staff available for caring for patients with class III obesity  [52] .

In the four case studies included in this review, it was found that patient comfort, dignity and appropriate care was compromised when appropriate equipment was unavailable. However, issues were resolved when staff collaborated with other disciplines to devise an overhead ceiling lift and obtained appropriate equipment [53]. Proactive efforts of staff enhanced collaboration with other health care staff [54] and resulted in the development of a daily schedule to establish a patient care routine [38]. In the four case studies included in this review, it was found that patient comfort, dignity and appropriate care were  compromised when appropriate equipment was unavailable. 

## Discussion

Nurses play a key role in the management of patients with obesity and their practice needs to be underpinned by best evidence. This scoping review identified a paucity of evidence to inform the nursing care of people with Class III obesity in acute care settings. Nurses are caring for patients across the BMI spectrum and the number of people admitted to acute care settings who are classified as obese is increasing. The availability of guidance to inform and support care  is vital if patient outcomes are to be optimised. It has been reported that nurses are reluctant to care for these patients, reporting weight bias [57, 58], and high risk of injury and compensation claims by staff have been reported [20, 59], with the risk of sustaining a musculoskeletal injury for both patients and staff is higher when the patient is classified as class III obese [20, 21]. Issues related to timely access of appropriate equipment dominated the findings. Inadequacies in the provision, access and resourcing of specialist equipment to care for patients, as well as the time taken to source the equipment, has been reported as a significant issue for staff [52, 60, 61]. Lack of adequate bariatric equipment accounted for the majority of clinical incidents reported in one study [41]. Lack of equipment was also associated with patient harm [45] [45]. Whilst there is a perception that patients who are obese have higher care needs [29], perceptions such as these have not been extensively validated. The identification of patients within the National Hospital Morbidity Database and outcomes related to length of stay, morbidity and readmission rates would provide a clearer picture and identify the need for change [62]. To ensure clinical staff deliver care based on best available evidence, it is essential to develop and make widely available, policies and procedures that focus on lifting protocols, lift teams, appropriate equipment and algorithms to promote safety and dignity. It is therefore recommended that an exploration into the many components of the patient journey is undertaken which can provide an evidence base in this area.

This review also found a lack of consistency within the literature relating to terminology and definitions used to define the level of obesity. Inconsistency can lead to confusion, inaccuracy, and a lack of transferability when developing protocols and systems within acute care and pose clinical risk to both patients and staff. It is recommended that a consistent approach to terminology should be adopted such as those defined by the WHO [3] which are widely accessible. Consistency in the application of terminology could make a significant impact on patient care.

It was found that there were variations in the definition of Class III obesity across the 14 studies, from no definition at all, definitions based on the Body Mass index (BMI) to whether weight exceeded equipment size (Table 4).

Evidence of organisational-wide innovations in the care of the Class III obese patient was limited. The creation of dedicated manual handling teams lead to a reduction in hospital-acquired pressure ulcers, staff injuries, health care costs and an increase in staff satisfaction [63]. Patient and staff satisfaction were increased when a safe patient handling coordinator was appointed to oversee policy and procedures and to provide education for nursing staff [64]. This  role included the coordination of patient care within the healthcare system, including planning and decision making with key stakeholders in different departments. This review found that nurse-led patient case conferences [40], ongoing moving and handling assessments [53] and streamlined admission processes to anticipate equipment needs [45] were opportunities to improve practice but there is a lack of evidence in the literature that these initiatives have been evaluated or widely implemented.

It is therefore recommended that an exploration into the many components of the patient journey is undertaken which can provide an evidence base in this area.

The concept of weight bias was not a major issue raised specifically in this review as this was not the main focus of it, but the presence of weight bias has been acknowledged within the general population, and within a range of health care providers including nurses [65–67]. This may be an area that requires further attention in future research to ensure that care is not compromised. There is limited evidence on interventions to reduce weight bias [65], but simulated educational techniques have shown promise [66, 67]. Inter professional research into methods to reduce weight bias and incorporating the patient’s voice have been called for [65].

One study explored the Class III obese patient experience during an acute care admission [29], the remainder focused on the care of patients already admitted to acute care settings. The findings reported that even though the admission was a planned one, the equipment needed was either not available or appropriate. While the evidence is limited, in the two case studies included in the review, the the findings were similar. One study in particular noted the delay in accessing appropriate equipment [52]. More evidence is needed to determine the patient experience across a range of contexts to inform and guide care.

### Limitations

The study was limited to primary studies published in English. It is not known if studies in languages other than English have similar results. We also excluded opinion and editorial texts which may discuss similar barriers and enablers to care.

## Conclusions

The areas of care reported as the most challenging for nurses when caring for patients with Class III obesity included wound management, mobilisation, maintaining dignity, comfort and safety. Specific guidelines that inform these aspects of care would support nurses to deliver optimum care and go some way to de-stigmatising the management of this population within acute care. There was minimal evidence of proactive planning to ensure the availability of well-educated staff, familiar and confident with the use of suitable equipment to assist with manual handling to prevent injury to both patient, staff, and to maintain patient dignity. What was evident in the literature was the inconsistency with terminology that defines this group of patients. This could lead to inaccurate application of guidelines when caring for this population.

As a recommendation arising from this review, it is suggested that consistency in the terminology of the classification of obesity, to ensure uniformity in the application of procedures and guidelines, increasing the safety of practice and patient care. A robust body of evidence which informs the unique nursing care needs of patients with Class III obesity is urgently required.

## Supplementary Information

**Additional file 1: Table A.** Logic Grid for Class III Obesity Scoping Review. **Table B.** Articles excluded with reasons.

## Data Availability

Data sharing is not applicable to this article as no datasets were generated or analysed during the current study.

## References

[1] Liodaki E, Senyaman Ö, Stollwerck PL, Möllmeier D, Mauss KL, Mailänder P, Stang F (2014). Obese patients in a burn care unit: a major challenge. Burns..

[2] Hruby A, Manson JE, Qi L, Malik VS, Rimm EB, Sun Q (2016). Determinants and consequences of obesity. Am J Public Health.

[3] World Health Organisation. Obesity and Overweight Fact Sheet: World Health Organistaion; 2020 [Available from: http://www.who.int/mediacentre/factsheets/fs311/en/.

[4] Sultana N, Afroz S, Tomalika N, Momtaz H, Kabir MH (2019). Prevalence of childhood obesity and undernutrition among urban school children in Bangladesh. J Biosoc Sci.

[5] Siddiqui MZ, Donato R (2016). Overweight and obesity in India: policy issues from an exploratory multi-level analysis. Health Policy Plan.

[6] Hammad SSMSRN, Berry DCPANPBCFF (2017). The child obesity epidemic in Saudi Arabia: a review of the literature. J Transcult Nurs.

[7] Korda RJ, Liu B, Clements MS, Bauman AE, Jorm LR, Bambrick HJ (2013). Prospective cohort study of body mass index and the risk of hospitalisation: findings from 246361 participants in the 45 and Up Study. Int J Obes (2005).

[8] Cowley SP, Leggett S (2010). Manual handling risks associated with the care, treatment and transportation of bariatric patients and clients in Australia. Int J Nurs Pract.

[9] Rolland-Cachera MF (2012). Towards a simplified definition of childhood obesity? A focus on the extended IOTF references. Pediatric obesity.

[10] French SA, Wall M, Corbeil T, Sherwood NE, Berge JM, Neumark-Sztainer D (2018). Obesity in adolescence predicts lower educational attainment and income in adulthood: the project EAT longitudinal study. Obesity..

[11] Jahangir E, De Schutter A, Lavie CJ (2014). Low weight and overweightness in older adults: risk and clinical management. Prog Cardiovasc Dis.

[12] Upadhyay J, Farr O, Perakakis N, Ghaly W, Mantzoros C (2017). Obesity as a disease. Med Clin.

[13] Kingston A, Byles J, Kiely K, Anstey K, Jagger C. The impact of smoking and obesity on disability-free life expectancy in older Australians. The journals of gerontology Series A, Biological sciences and medical sciences. 2020. 10.1093/gerona/glaa290 PMC8202145 33249489

[14] Lung T, Jan S, Tan EJ, Killedar A, Hayes A (2019). Impact of overweight, obesity and severe obesity on life expectancy of Australian adults. Int J Obes.

[15] Lartey S, Si L, Lung T, Magnussen CG, Boateng GO, Minicuci N, et al. Impact of overweight and obesity on life expectancy, quality-adjusted life years and lifetime costs in the adult population of Ghana. BMJ global health. 2020;5(9). 10.1136/bmjgh-2020-003332 PMC7526271 32994229

[16] Imber DA, Pirrone M, Zhang C, Fisher DF, Kacmarek RM, Berra L (2016). Respiratory Management of Perioperative Obese Patients. Respir Care.

[17] Sauceda T, Falco K (2014). The bariatric patient and an unsupportive healthcare environment: An ethics analysis. Am J Safe Patient Handl Move.

[18] Winfield RD (2014). Caring for the critically ill obese patient. Nutr Clin Pract.

[19] Dambaugh LA, Ecklund MM (2016). Progressive Care of Obese Patients. Crit Care Nurse.

[20] McClean K, Cross M, Reed S (2021). Risks to Healthcare Organizations and Staff Who Manage Obese (Bariatric) Patients and Use of Obesity Data to Mitigate Risks: A Literature Review. J Multidisc Healthc [Internet].

[21] Choi SD, Brings K (2016). Work-related musculoskeletal risks associated with nurses and nursing assistants handling overweight and obese patients: a literature review. Work..

[22] Richardson A, Gurung G, Derrett S, Harcombe H (2019). Perspectives on preventing musculoskeletal injuries in nurses: a qualitative study. Nurs Open.

[23] Tizer K (2007). Extremely obese patients in the healthcare setting: patient and staff safety. J Ambul Care Manage.

[24] D'Arcy Y (2015). Managing pain in obese patients. Nursing..

[25] Berrios LA (2016). The ABCDs of managing morbidly obese patients in intensive care units. Crit Care Nurse.

[26] Cowdell F, Radley K (2014). What do we know about skin-hygiene care for patients with bariatric needs? Implications for nursing practice. J Adv Nurs.

[27] Phibbs SC, Faith J, Thorburn S (2017). Patient-centred communication and provider avoidance: does body mass index modify the relationship?. Health Educ J.

[28] Koball AM, Mueller PS, Craner J, Clark MM, Nanda S, Kebede EB, Grothe KB (2018). Crucial conversations about weight management with healthcare providers: patients' perspectives and experiences. Eat Weight Disord.

[29] Hales C, Curran N, de Vries K (2018). Morbidly obese patients’ experiences of mobility during hospitalisation and rehabilitation: a qualitative descriptive study. Nurs Prax N Z.

[30] Ladd SB, Ekanem UI, Caffrey J (2018). 540 A Systematic Review of Pressure Ulcers in Burn Patients: Risk Factors, Demographics, and Treatment Modalities. J Burn Care Res.

[31] Tellson A, Qin H, Erwin K, Houston S (2017). Efficacy of acute care health care providers in cardiopulmonary resuscitation compressions in normal and obese adult simulation manikins. Proc (Baylor Univ Med Cent).

[32] Peters MDJ, Godfrey CM, McInerney P, Munn Z, Tricco AC, Khalil H. Scoping reviews (2020 version). In: Aromataris E, Munn Z, editors. JBI manual for evidence synthesis2020.

[33] Tricco AC, Lillie E, Zarin W, O'Brien KK, Colquhoun H, Levac D, Moher D, Peters MDJ, Horsley T, Weeks L, Hempel S, Akl EA, Chang C, McGowan J, Stewart L, Hartling L, Aldcroft A, Wilson MG, Garritty C, Lewin S, Godfrey CM, Macdonald MT, Langlois EV, Soares-Weiser K, Moriarty J, Clifford T, Tunçalp Ö, Straus SE (2018). PRISMA extension for scoping reviews (PRISMA-ScR): checklist and explanation. Ann Intern Med.

[34] Peters MDJ, Marnie C, Tricco AC, Pollock D, Munn Z, Alexander L, McInerney P, Godfrey CM, Khalil H (2020). Updated methodological guidance for the conduct of scoping reviews. JBI Evid Synth.

[35] Pollock D, Tricco AC, Peters MDJ, McInerney PA, Khalil H, Godfrey CM, et al. Methodological quality, guidance, and tools in scoping reviews a scoping review protocol. JBI Evid Synth. 2021;Ahead of Print. 10.11124/JBIES-20-00570 34446668

[36] Munn Z, Peters MDJ, Stern C, Tufanaru C, McArthur A, Aromataris E (2018). Systematic review or scoping review? Guidance for authors when choosing between a systematic or scoping review approach. BMC Med Res Methodol.

[37] Moher D, Liberati A, Tetzlaff J, Altman DG, Group TP (2009). Preferred reporting items for systematic reviews and Meta-analyses: the PRISMA statement. PLoS Med.

[38] Broome CA, Ayala EM, Georgeson KA, Heidrich SM, Karnes K, Wells JB (2015). Nursing Care of the Super Bariatric Patient: challenges and lessons learned. Rehabilitation Nursing.

[39] Ecklund MM (2004). Meeting the nutritional needs of the bariatric patient in acute care. Crit Care Nurs Clin North Am.

[40] Holland DE, Krulish YA, Reich HK, Roche JD (2001). How to creatively meet care needs of the morbidly obese. Nurs Manag.

[41] Booth CMA, Moore CE, Eddleston J, Sharman M, Atkinson D, Moore JA (2011). Patient safety incidents associated with obesity: a review of reports to the National Patient Safety Agency and recommendations for hospital practice. Postgrad Med J.

[42] Drake DJ, Baker G, Engelke MK, McAuliffe M, Pokorny M, Swanson M (2008). Challenges in caring for the morbidly obese: differences by practice setting. Southern Online J Nurs Res.

[43] Gardner L, Pagano M. Skin Integrity, Immobility, and Pressure Ulcers in Class III Obese Patients Pennsylvania Patient Safety Advisory. 2013a;10(2):50–4 Retrieved from http://www.patientsafetyauthority.org/ADVISORIES/AdvisoryLibrary/2013/Jun;10(2)/Pages/50.aspx.

[44] Gardner L, Gibbs C (2013). Class III obese patients: Is your hospital equipped to address thier needs?. Pennsylvania Patient Safe Advis.

[45] Gardner LA, Gibbs C (2013). Class III obese patients: Is your hospital equipped to address thier needs?. Pennsylvania Patient Safety Advisory.

[46] Gardner L, Pagano M (2013). Class III Obese Patients: The Effect of Gait and Immobility on Patient Falls. Pennsylvania Patient Safety Advisory.

[47] Hignett S, Chipchase S, Tetley A, Griffiths P. Risk Assessment and Process Planning for Bariatric Patient Handling Pathways. Norwich, UK: Health and Safety Executive,: http://www.hse.gov.uk/research/rrpdf/rr573.pdf; 2007.

[48] Rose MA, Baker G, Drake DJ, Engelke M, McAuliffe M, Pokorny M, Pozzuto S, Swanson M, Waters W, Watkins F (2007). A comparison of nurse staffing requirements for the Care of Morbidly Obese and non-Obese Patients in the acute care setting. Bariatric Nurs Surg Patient Care.

[49] Drake D, Dutton K, Engelke M, McAuliffe M, Rose MA (2005). Challenges that nurses face in caring for morbidly obese patients in the acute care setting. Surg Obes Relat Dis.

[50] Rose MA, Pokorny M, Waters W, Watkins F, Drake DJ, Kirkpatrick M (2010). Nurses' perceptions of safety concerns when caring for morbidly obese patients. Bariatric Nurs Surg Patient Care.

[51] Rose MA, Pokorny M, Waters W, Watkins F, Drake DJ, Kirkpatrick M (2010). Nurses' perceptions of safety concerns when caring for morbidly obese patients. Bariatr Nurs Surg Patient Care.

[52] Dockrell S, Hurley G (2021). Moving and handling care of bariatric patients: a survey of clinical nurse managers. J Res Nurs.

[53] Palmer R (2004). Moving and handling bariatric patients safely: a case study. Int J Ther Rehabil.

[54] Ecklund MM, Kurlak SA (2004). Caring for the bariatric patient with obstructive sleep apnea. Crit Care Nurs Clin.

[55] Drake DJ, Swanson M, Baker G, Pokorny M, Rose MA, Clark-Reed L, Waters W, Watkins FR, Engelke MK (2010). The association of BMI and Braden total score on the occurrence of pressure ulcers. Journal of Wound, Ostomy and Continence Nursing.

[56] Gardner LA, Pagano M (2013). Class III Obese Patients: The Effect of Gait and Immobility on Patient Falls. Pennsylvania Patient Safety Advisory.

[57] Robstad N, Westergren T, Siebler F, Söderhamn U, Fegran L (2019). Intensive care nurses' implicit and explicit attitudes and their behavioural intentions towards obese intensive care patients. J Adv Nurs (John Wiley & Sons, Inc).

[58] Pervez H, Ramonaledi S (2017). Nurses' attitudes towards obese patients: a review of the literature. Nurs Times.

[59] McClean K, Cross M, Reed S (2021). Evaluating the effectiveness of a clinical practice intervention in increasing obesity data recording at a Western Australian country health service hospital: a quasi-experimental controlled trial. J Multidiscip Healthc.

[60] Lumley E, Homer CV, Palfreyman S, Shackley P, Tod A (2015). M. a qualitative study to explore the attitude of clinical staff to the challenges of caring for obese patients. J Clin Nurs.

[61] Yüksel S, Kettaş E, Randa S (2017). Are nurses willing to provide care to obese surgical patients?. Bariatr Surg Pract Patient Care.

[62] Dvd Z, Pointer S, Harrison JE, Australia. Department of H, ageing, Australian Institute of H (2013). Obesity and injury in the National Hospital Morbidity Database.

[63] Walden CM, Bankard SB, Cayer B, Floyd WB, Garrison HG, Hickey T, Holfer LD, Rotondo MF, Pories WJ (2013). Mobilization of the obese patient and prevention of injury. Ann Surg.

[64] Thomas SA, Rickabaugh B. Bariatric nurse coordinator: carving out a new role in bariatrics. Bariatric Nursing and Surgical Patient Care. 2008;3(1). p. 63-72

[65] Alberga AS, Pickering BJ, Alix Hayden K, Ball GDC, Edwards A, Jelinski S, Nutter S, Oddie S, Sharma AM, Russell-Mayhew S (2016). Weight bias reduction in health professionals: a systematic review weight bias reduction in health professionals. Clin Obes.

[66] Boyle SL, Janicke DM, Robinson ME, Wandner LD (2019). Using virtual human technology to examine weight Bias and the role of patient weight on student assessment of pediatric pain. J Clin Psychol Med Settings.

[67] Hales C, Gray L, Russell L, MacDonald C (2018). A qualitative study to explore the impact of simulating extreme obesity on health care Professionals' attitudes and perceptions. Ostomy Wound Manage.

