# Supplementary material for: The nursing care of people with class III obesity in an acute care setting: a scoping review
Source: BMC Nurs. 2022 Jan 28;21:33. doi: 10.1186/s12912-021-00760-7 (PMC8796636; doi:10.1186/s12912-021-00760-7)
Supplement: Supplementary file 1 — Additional file 1: Table A. Logic Grid for Class III Obesity Scoping Review. Table B. Articles excluded with reasons. [file 12912_2021_760_MOESM1_ESM.zip › Appendix A Supplementary Material.docx]

**Table A Logic Grid for Class III Obesity Scoping Review**

| **Logic Grid aligned with PCCs of the review question and search strategy** | | | |
| --- | --- | --- | --- |
| **Population** | **Concept** | **Context** | **Studies** |
| **People with Class III obesity** | **Nursing care** | **Acute care settings** | **Primary studies, case reports** |
| **Logic Grid with identified key words and search terms** | | | |
| **Population** | **Concept** | **Context** | **Studies** |
| morbid obesity | patient care | hospital care | case reports |
| bariatric patients | best practice care | in-patient care | primary research studies |
| bariatric* |  |  |  |
| morbidly obese | patient handling | hospital policies |  |
| obes* | Safe patient handling | acute care setting* |  |
| morb* obes* | nursing care |  |  |
| Limiters English Language, Academic articles, since 1980, All Adult, Inpatients  First Search in CINAHL +  (((obesity, morbid or Bariatric Patients OR morbidly obese OR Obes* OR Morbd* Obes*) AND (nursing care) OR (patient care) OR (best practice care) OR (patient handling) OR (Acute care settings) OR (hospital care) OR( in-patient care) OR (hospital polic*)))  First search yielded 30 results. | | | |

Appendix Figure 1

| JBI Critical Appraisal Checklist for Case Reports | |
| --- | --- |
| Q1 | Were patient’s demographic characteristics clearly described? |
| Q2 | Was the patient’s history clearly described and presented as a timeline? |
| Q3 | Was the current clinical condition of the patient on presentation clearly described? |
| Q4 | Were diagnostic tests or assessment methods and the results clearly described? |
| Q5 | Was the intervention(s) or treatment procedure(s) clearly described? |
| Q6 | Was the post-intervention clinical condition clearly described? |
| Q7 | Were adverse events (harms) or unanticipated events identified and described? |
| Q8 | Does the case report provide takeaway lessons? |

| JBI Critical appraisal Checklist for Descriptive/Case Series | |
| --- | --- |
| Q1 | Were there clear criteria for inclusion in the case series? |
| Q2 | Was the condition measured in a standard, reliable way for all participants included in the case series? |
| Q3 | Were valid methods used for identification of the condition for all participants included in the case series? |
| Q4 | Did the case series have consecutive inclusion of participants? |
| Q5 | Did the case series have complete inclusion of participants? |
| Q6 | Was there clear reporting of the demographics of the participants in the study? |
| Q7 | Was there clear reporting of clinical information of the participants? |
| Q8 | Were the outcomes or follow up results of cases clearly reported? |
| Q9 | Was there clear reporting of the presenting site(s)/clinic(s) demographic information? |
| Q10 | Was statistical analysis appropriate? |

| JBI Critical Appraisal Checklist for Interpretive and Critical Research | |
| --- | --- |
| Q1 | Is there congruity between the stated philosophical perspective and the research methodology? |
| Q2 | Is there congruity between the research methodology and the research question or objectives? |
| Q3 | Is there congruity between the research methodology and the methods used to collect data? |
| Q4 | Is there congruity between the research methodology and the representation and analysis of data? |
| Q5 | Is there congruity between the research methodology and interpretation of results? |
| Q6 | Is there a statement situating the researcher culturally or theoretically? |
| Q7 | Is the influence of the researcher on the research and vice versa addressed? |
| Q8 | Are participants and their voices adequately represented? |
| Q9 | Is the research ethical according to current criteria, or for recent studies, is there evidence of ethical approval by an appropriate body? |
| Q10 | Do the conclusions drawn in the research report flow from the analysis or interpretation of data? |

| JBI Critical Appraisal Checklist for Analytical Cross Sectional Studies | |
| --- | --- |
| Q1 | Were the criteria for inclusion in the sample clearly defined? |
| Q2. | Were the study subjects and the setting described in detail? |
| Q3. | Was the exposure measured in a valid and reliable way? |
| Q4. | Were objective, standard criteria used for measurement of the condition? |
| Q5. | Were confounding factors identified? |
| Q6. | Were strategies to deal with confounding factors stated? |
| Q7. | Were the outcomes measured in a valid and reliable way? |
| Q8. | Was appropriate statistical analysis used |
