# Supplementary material for: The nursing care of people with class III obesity in an acute care setting: a scoping review
Source: BMC Nurs. 2022 Jan 28;21:33. doi: 10.1186/s12912-021-00760-7 (PMC8796636; doi:10.1186/s12912-021-00760-7)
Supplement: Supplementary file 1 — Additional file 1: Table A. Logic Grid for Class III Obesity Scoping Review. Table B. Articles excluded with reasons. [file 12912_2021_760_MOESM1_ESM.zip › Table B .docx]

| **Table B Articles excluded with reasons** | | |
| --- | --- | --- |
| **Author/Date** | **Article Title** | **Reason for excluding** |
| Baldwin et al, 2011 | Does morbid obesity negatively affect the hospital course of patients undergoing treatment of closed, lower-extremity diaphyseal long-bone fractures?  Orthopaedics, 34(1), 18-18. | Outcomes focused |
| Batsis et al, 2009 | Impact of body mass on hospital resource use in total hip arthroplasty. Public Health Nutrition, 12(8), 1122-1132. | Not nursing care |
| Cheung et al, 2006 | Failure modes and effects analysis: minimising harm to our bariatric patients. Bariatric Nursing and Surgical Patient Care 1 (2) 107-114 | Not nursing care |
| Coen et al,  2011 | The results of a national survey regarding nutritional care of obese burn patients. Journal of Burn Care & Research, 32(5), 561-565. | Not nursing care |
| Corbyn & Rush, 2010 | Challenges of wound management in bariatric patients. Wounds UK, 6(4), 62-71. | Community care |
| Cowley & Leggett, 2010 | Manual handling risks associated with the care, treatment and transportation of bariatric patients and clients in Australia. International Journal of Nursing Practice, 16(3), 262-267. | Not nursing focused |
| Drake et al, 2010 | The association of BMI and Braden total score on the occurrence of pressure ulcers. Journal of Wound, Ostomy and Continence Nursing, 37(4), 367-371. | Prevention, not nursing care |
| Evanoff et al, 2003 | Reduction in injury rates in nursing personnel through introduction of mechanical lifts in the workplace.  American Journal of Industrial Medicine, 44(5), 451-457 | Not Class III Obesity specific |
| Harris, 2008 | Nursing care of the morbidly obese patient. Nursing Made Incredibly Easy 6(3), 34-43. | Opinion |
| Hignett & Griffiths , 2009 | Risk factors for moving and handling bariatric patients. Nursing Standard 24(11), 40-48. | Same data from previous Hignett et al, 2007 study |
| Hignett & Griffiths, 2009 | Manual handling risks in the bariatric (obese) patient pathway in acute sector, community and ambulance care and treatment. Work, 33(2), 175-180. | Same data from previous Hignett et al, 2007 study |
| Kramer, 2004 | WOC Nurses as Advocates for Patients Who Are Morbidly Obese: A Case Study Promoting the Use of Bariatric Beds. Journal of Wound Ostomy & Continence Nursing, 31(6), 379-384. | More opinion than case report |
| Muir & Gerlach, 2003 | Reducing the risks in bariatric patient handling: The crucial need for ensuring worker safety while treating all patients with respect prompted one healthcare facility to develop a program targeted toward management of the obese patient. The Canadian Nurse, 99(8), 29-33. | Opinion |
| Nowicki et al, 2009 | Changing the mindset: An inter-disciplinary approach to management of the bariatric patient. Collegian, 16(4), 171-175. | Opinion |
| Payvar et al, 2013 | In-hospital outcomes of percutaneous coronary interventions in extremely obese and normal-weight patients: findings from the NCDR (National Cardiovascular Data Registry). Journal Of The American College Of Cardiology, 62(8), 692-696 | Outcomes not nursing care |
| Randall et al, 2009 | Expanded Occupational Safety and Health Administration 300 log as metric for bariatric patient-handling staff injuries. Surgery for obesity and related diseases: official journal of the American Society for Bariatric Surgery, 5(4), 463-468. | Not nursing care |
| Roberts & Bates, 1992 | The use of the Body Mass Index in studies of abdominal wound infection. Journal of Hospital Infection, 20(3), 217-220. | Outcomes not nursing care |
| Rose et al, 2006 | Nurse Staffing Requirements for Care of Morbidly Obese Patients in the Acute Care Setting115-121. Bariatric Nursing and Surgical Patient Care, 1(2). 115-121 | Data from another study |
| Swanson et al, 2011 | Braden subscales and their relationship to the prevalence of pressure ulcers in hospitalized obese patients. Bariatric Nursing and Surgical Patient Care, 6(1). 21-23 | Prevalence study |
| Vellinga et al, 2008 | Length of stay and associated costs of obesity related hospital admissions in Ireland.  BMC Health Services Research, 8(1), 88. doi: 10.1186/1472-6963-8-88 | Outcomes not nursing care |
| Westerly & Dabbagh, 2011 | Morbidity and mortality characteristics of morbidly obese patients admitted to hospital and intensive care units. Journal of Critical Care, 26(2), 180-185 | Not nursing care |
| Zizza et al, 2004 | Length of Hospital Stays Among Obese Individuals. American Journal of Public Health, 94(9), 1587-1591. | Not nursing focused |
| Zuzelo & Seminara, 2006 | Influence of registered nurses' attitudes toward bariatric patients on educational programming effectiveness. Journal of Continuing Education in Nursing, 37(2), 65-73. | Outcomes not nursing care |
